# Cellular Responses Evoked by Different Surface Characteristics of Intraosseous Titanium Implants

**DOI:** 10.1155/2015/171945

**Published:** 2015-02-12

**Authors:** Liviu Feller, Yusuf Jadwat, Razia A. G. Khammissa, Robin Meyerov, Israel Schechter, Johan Lemmer

**Affiliations:** ^1^Department of Periodontology and Oral Medicine, Sefako Makgatho Health Sciences University, Pretoria 0204, South Africa; ^2^Schulich Faculty of Chemistry, Technion-Israel Institute of Technology, 32000 Haifa, Israel

## Abstract

The properties of biomaterials, including their surface microstructural topography and their surface chemistry or surface energy/wettability, affect cellular responses such as cell adhesion, proliferation, and migration. The nanotopography of moderately rough implant surfaces enhances the production of biological mediators in the peri-implant microenvironment with consequent recruitment of differentiating osteogenic cells to the implant surface and stimulates osteogenic maturation. Implant surfaces with moderately rough topography and with high surface energy promote osteogenesis, increase the ratio of bone-to-implant contact, and increase the bonding strength of the bone to the implant at the interface. Certain features of implant surface chemistry are also important in enhancing peri-implant bone wound healing. It is the purpose of this paper to review some of the more important features of titanium implant surfaces which have an impact on osseointegration.

## 1. Introduction

An ideal implant surface should exhibit both osseoconductive and osseoinductive properties, promoting peri-implant bone wound healing and consequently the formation of well-organized mature bone of high mineral and trabecular density with a high proportion of bone-to-implant contact, which will withstand the stress generated on the osseointegrated implant by occlusal forces [1, 2]. The degree of roughness of the implant surface and surface chemistry, topography, and energy/wettability affect cellular responses such as cell adhesion, proliferation, differentiation, and migration, thus influencing peri-implant endosseous healing [3–7].

In general, adhesion of cells to a biomaterial is mediated by several mechanisms. These mechanisms include specific interactions between cell surface receptors and specific ligand molecules which are adsorbed to, deposited on, or secreted over the biomaterial; nonspecific forces such as van der Waal and electrostatic forces; and mechanical anchorage to the micro- and nanotopographical structures of the implant surface [8]. Cells can recognise and react differently to different surface characteristics of an implant. Cell populations in contact with such different implant surfaces exhibit gene expression, metabolic activities, and phenotypic characteristics specific to the surface, thus influencing peri-implant bone wound healing [9]. Bone marrow progenitor cells and osteogenic cells in response to different implant surface characteristics will express the genes associated with sequential biological events of osteogenesis [10, 11].

## 2. Some Biological Events Associated with Interactions between Cells and Biomaterial Surfaces

Cells interact with the protein-conditioned layer on the implant surface, and although the chemical and physical characteristics of this layer may be different from those of the implant surface, the biological interactions are mainly dictated by the physicochemical characteristics of the implant itself [12].

As a cell approaches the titanium (biomaterial) surface, cell attachment occurs first, sometimes followed by cell adhesion. Both processes are primarily driven by the energy and wettability of the surface [12]. While the former is merely a function of the implant physical and chemical characteristics, the latter is governed by both the implant and by its bioenvironment.

The surface energy may be defined as the excess energy at the surface of a material compared to the bulk [13]. Surface energy quantifies the disruption of intermolecular bonds that occur when a surface is created. In most cases, surfaces are less energetically favorable than the bulk, which means that the molecules on the surface have more energy compared with the molecules in the bulk [14]. This extra energy provides the driving force for the adhesion to the surrounding tissues. In other words, an active implant surface provides the required conditions for starting the desired interaction with the cellular environment.

On the other hand, wettability describes the balance between the intermolecular interactions when a solid surface and a liquid are brought together [15]. It describes the ability of a liquid to maintain contact with a solid surface. The wettability is determined by a balance between adhesive and cohesive forces. The adhesive forces between a liquid and a solid cause a liquid drop to spread across the surface and the cohesive forces within the liquid cause the drop to ball up and minimize contact with the surface. Therefore, in an interaction of a liquid drop with a solid surface, the wettability can be calculated from the contact angle that is formed between the drop and the surface. In this case, the contact angle provides an inverse measure of the wettability [16]. The actual interactions of an implant with its microenvironment are much more complicated and cannot be described by such a simplistic model. Nevertheless, these general considerations are still valid [17].

**Figure 1:**
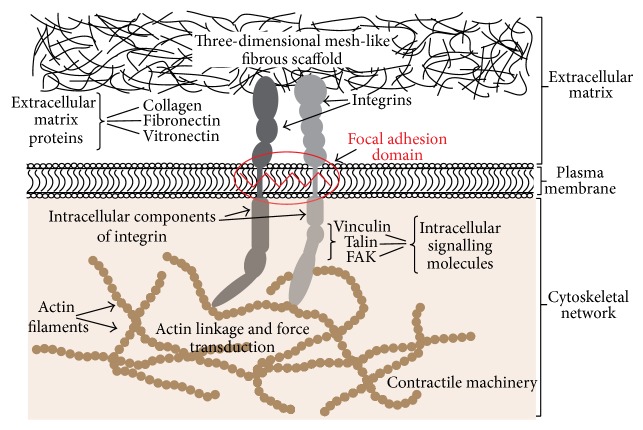
Extracellular matrix proteins such as collagen, fibronectin, and vitronectin bind to integrins stimulating intracellular signaling pathways, which modulate cell function, and mediating cell adhesion, migration, differentiation, and proliferation.

Cell attachment occurs when the cell is within 2–5 nanometres of the biomaterial surface and is mediated by electrostatic forces. On the other hand cell adhesion occurs only if the cell membrane comes into direct contact with the biomaterial surface, when atomic-level interactions can be established [12]. Initially, cell adhesion to the titanium surface is mediated by covalent, ionic, hydrogen, or charge-transfer bonds [12]. For example, electron donor sites on the surface of an osteoblast interact with electron acceptor sites on the titanium oxide surface resulting in osteoblast adhesion and differentiation [18]. Later, cell adhesion is mediated by multifunctional cellular structures consisting of a complex network of transplasma membrane integrins and cytoplasmic proteins linking the extracellular matrix (ECM) to the cytoskeleton, and such adhesion is termed focal adhesion [19]. ECM ligands that interact with the extracellular domain of integrins include fibronectin, vitronectin, and collagen. The intracellular part of the integrin interacts with the actin cytoskeleton and other proteins of the focal adhesion domain [20–23]. Thus, focal adhesions provide a vehicle for cross-talk between the ECM and the cell, on the one hand regulating ECM protein assembly and remodeling and on the other hand regulating cell adhesion, migration, proliferation, differentiation, and apoptosis (Figure 1) [22, 24].

The ECM is a complex assembly of molecules that interact with one another [25] creating the physical microenvironment necessary for the cell to survive and to function, for cell anchorage, and for providing a tissue scaffold for cell migration [22]. The molecular composition, the architecture of the three-dimensional structure of the ECM, and its mechanical properties play essential roles in mediating cellular responses [22, 26].

The ECM is composed of three-dimensional mesh-like fibrous scaffold that generates mechanical forces. Integrins in focal adhesions act as mechanoreceptors, activating intracellular signal transduction pathways which generate biochemical cellular responses (Figure 1) [22, 26]. Furthermore, ECM-induced mechanical stimulation brings about maturation of existing focal adhesions and formation of new focal adhesions, with a consequent increase in the strength and the rigidity of the connection between the integrins and the cytoskeleton. These phenomena affect cell phenotype and influence cell adhesion and migration [26].

Mechanically, stressed matrices are associated with increased cell proliferation, while unstressed matrices are associated with downregulation of cellular proliferation. When the matrix is stressed, the cells develop isometric tension which is equal to the mechanical tensional force exerted upon them by the ECM [24]. These forces regulate cellular architecture and activate cellular transcription factors which in turn determine gene expression [26].

The isometric tension within the cell may in turn change the configuration of ECM proteins in such a way that specific integrin recognition molecules of the ECM proteins become exposed, triggering integrin-induced cell activation as outlined above. In a similar manner, specific topographic features of the ECM in relation to its three-dimensional structure may induce stretching of specific proteins, exposing integrin recognition sites and favouring cell activation [24]. In addition, the mechanical- and topographical-induced configurational changes in ECM proteins attract to the focal adhesion domain specific integrins that further facilitate ECM-cell interactions [26].

On the other hand, once cells have established focal adhesions to the ECM, they transfer tension that is generated by their actin cytoskeleton to extracellular fibronectin, exposing cryptic sites for polymerization, mediating fibronectin fibrillogenesis which in turn mediates patterning of collagen fibres, thus promoting the organization of a three-dimensional ECM. Reciprocally as described above, ECM mediates cell activities including cell attachment, proliferation, differentiation, migration, and apoptosis [22, 24].

However, cells in a particular three-dimensional matrix interact not with a single protein but with numerous proteins and their three-dimensional matrix adhesions will activate multiple intracellular signal transduction pathways, which regulate gene transcription culminating in ECM-induced cell proliferation, migration, and apoptosis [26]. Extracellular matrices of different biophysical environments will activate different intracellular signal transduction pathways, thus eliciting varying cell responses [26]. In addition, cells can also be stimulated by the ECM through other mechanisms including shear stress from flowing fluids, chemical signals, and stretch-activated ion channels [22]. Although cell adhesion to ECM is largely mediated by focal adhesions, cell adhesion to ECM is also mediated by glycosaminoglycans such as hyaluronan [22].

Cells also interact with the ECM through cytoplasmic protrusions and filopodia, which probe and sense the topography of extracellular structures in contact with the cell, bringing about adjustment in cell shape and alignment for optimal adhesion to extracellular structures and allowing for cell migration and differentiation [20, 21, 27]. For example, complex surface topography, on both the micrometer and nanometer scales, promotes osteoblast adhesion and differentiation and affects osteoblast morphology [18]. Cell adhesion molecules, integrins, and cadherins are found on the filopodia, forming an initial adhesion site. Subsequently, other adhesion molecules including talin and paxillin are recruited to the initial site and are involved in the maturation of focal adhesions [27].

It appears that implant surface nanotopography induces expression of specific integrin subunits and induces synthesis of focal adhesion proteins, thus promoting osteoblast adhesion and migration. Furthermore, specific nanostructure-induced cell elongation can elicit cytoskeletal stress resulting in rapid selective osteoblastic differentiation of osteogenic cells [20, 21].

## 3. Properties of Titanium Biomaterials

**Figure 2:**
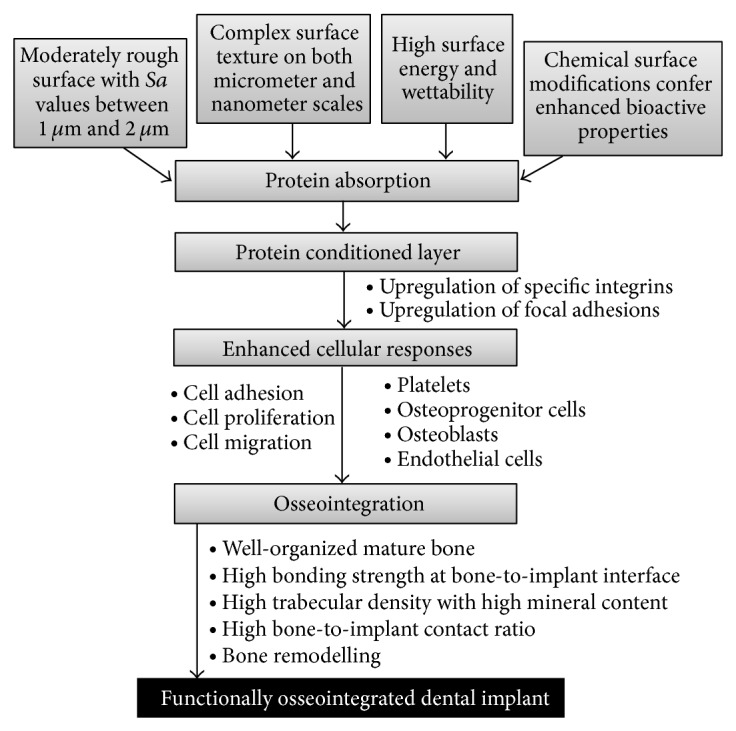
A flow chart showing how surface roughness, energy/wettability, and chemistry promote osseointegration of titanium dental implants.

Bioactive properties of titanium implants particularly the surface chemistry, surface topography, and surface energy/wettability affect the quality and the extent of osseointegration of the implant [3, 6, 28]. It is difficult to determine the relative effect of each of the surface properties of a titanium implant on the process of osseointegration, because the properties are interdependent (Figure 2) [29].

Surface topography describes the degree and the pattern of the roughness of the surface [29, 30]. A moderately rough surface promotes osteoblastic differentiation and osteogenesis on both micrometer and nanometer scales and increases the bone-to-implant contact ratio thus enhancing biomechanical interlocking (Figure 2) [6, 9, 28, 31]. However, the rougher the implant surface is, the more readily it accumulates bacterial plaques and the greater the risk of peri-implantitis will be. Therefore, the roughness of the implant surface should strike a balance between promoting favourable biological responses and avoiding detrimental plaque accumulation. Surface roughness of Sa values between 1 *μ*m and 2 *μ*m appears to be optimal [30, 32].

Implants with high surface energy are bioactive and will adsorb microenvironmental proteins [33]. Although surface energy is an important factor in mediating cellular activities, the surface energy of a smooth titanium implant is not sufficient to significantly promote osteogenic activities. However, the energy of a moderately rough titanium surface with complex micrometer and nanometer topography is sufficient to induce an osteogenic effect (Figure 2) [33].

The chemical composition of the biomaterial and the degree of roughness of its surface determine the surface energy and hence the wettability [33, 34]. Surface wettability in turn dictates biological responses favouring binding of proteins and consequently cell attachment, proliferation, and differentiation [9, 12, 20, 31, 33].

Immediately after insertion of an implant into bone, a process of chemical modification starts at the implant surface. This process is related to the exposure of the implant surface to an electrolytic environment and to ionic exchange with the surrounding tissues and fluids. This process can mediate differential adsorption and conformation of proteins which play an important role in platelet adhesion to the implant surface and in platelet activation [3, 35], driving the early events of peri-implant bone wound healing. Surface chemistry-dependent conformational changes in adsorbed proteins influence cellular activities. For example, specific structural changes in adsorbed fibronectin differentially modulate the expression of integrins on osteoblasts; integrins in turn regulate focal adhesion and intracellular signaling pathways [36].

Osseointegration is enhanced by the acquisition of bioactive properties by the chemical modification of an implant surface which promotes bonding with the peri-implant tissue during bone wound healing. However, it is uncertain whether such chemical modifications* per se* increase the implant anchorage by chemical bonds, or whether it is the increase in the microroughness on the nanometer scale or in the energy of the microrough surface that increases the strength of the implant-to-bone bonding [30, 37].

Anodization of the titanium implant surface changes the chemistry and the topography of the native titanium surface, resulting in a substantial thickness of the titanium oxide, with a porous topography and with a complex structure at both micrometer and nanometer scales [2, 7, 29, 38]. This may increase the bioactive properties of the surface, favouring osseointegration. The bone-titanium oxide interface displays continuous exchange of ions, promoting bone mineral precipitation on the implant surface [30, 31].

Hydroxylation/hydration of the titanium oxide layer increases the wettability of the implant surface promoting differentiation of osteogenic cells in the peri-implant microenvironment, increases implant-to-bone contact ratio, and improves the anchorage of the implant in bone [11, 34, 39].

Bone tends to grow into pores in the surface of a biocompatible implant material and this interlocking increases the anchorage of a porous implant surface, the strength of the anchorage being dependent on the degree of porosity (number of pores per unit area) and on the size of the pores [40]. However, the larger the pores are and the greater the number of pores is, the thinner the titanium septa will be between the pores so the structural integrity of the implant surface is decreased in proportion to the increase in number and size of the pores. This is a critical consideration in striking a balance between the degree of osseointegration and the load bearing capacity of the porous implant surface [41].

## 4. Osteogenesis in relation to the Topography of the Implant Surface

Immediately after implant insertion there is a greater degree of attachment of fibrin to the increased surface area of a moderately rough implant than to a smooth implant surface, thus enhancing the adhesion of a stable blood clot with the formation of a three-dimensional provisional fibrin matrix which serves as an osseoconductive scaffold for differentiating osteogenic cells migrating to the implant surface [42] and for the ingrowth of new blood vessels [39].

**Figure 3:**
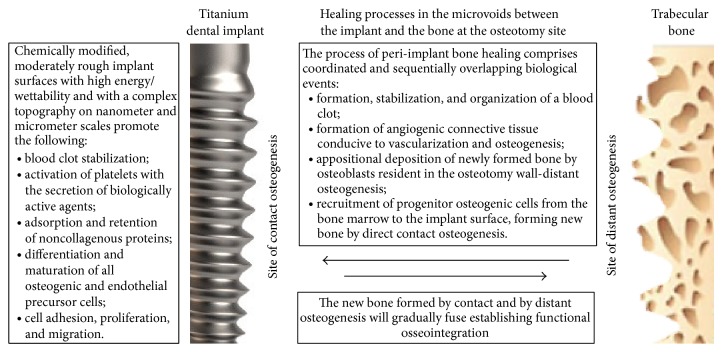
Early biological events of peri-implant bone healing include clot formation and immunoinflammatory responses. The platelets and the cells in the microenvironment produce cytokines and growth factors such as platelet derived growth factor, fibroblast growth factor, insulin growth factor, vascular endothelial growth factor, bone morphogenic proteins, interleukin-1*β*, and tumour necrosis factor *α*, some of which also recruit osteogenic precursor cells and subsequently stimulate their differentiation into osteoblasts. The fibrin based matrix of the clot is an osseoconductive medium that promotes the migration of some of the osteoprogenitor cells and endothelial progenitor cells to the implant surface. The osteoprogenitor cells and the vascular progenitor cells then differentiate into osteoblast depositing extracellular matrix which subsequently mineralizes and into endothelial cells which drive the process of neovascularization. Initially osteoblasts secrete noncollagenous bone proteins which attach to the protein conditioned layer of the implant surface. Later osteoblasts secrete collagenous precursor proteins that subsequently undergo mineralization [7].

Moderately rough implant surfaces not only favour blood clot stabilization but also promote activation of platelets [43, 44], which produce biological mediators including platelet derived growth factor, tumour growth factor *β*, insulin growth factor, and cytokines. Growth factors are also released from injured blood vessels and bone matrix in response to the bone drilling for implant insertion. In concert, these cytokines and growth factors accelerate the recruitment and stimulate the differentiation of both progenitor mesenchymal cells from the bone marrow in the peri-implant osteotomy walls and pericytes from blood vessel walls (Figure 3) [7, 43, 45].

It appears that bone formation on the surface of an artificial biomaterial is very similar to the bone formation that occurs during physiological remodeling. Physiologically, bone remodeling starts with osteoclastic bone resorption characterized by dissolution of the inorganic matrix of the bone followed by enzymatic degradation of the organic component of the matrix, creating a complex three-dimensionally structured surface. Osteoblasts then secrete noncollagenous proteins which permeate the surface irregularities and undergo mineralization, forming a thin layer of noncollagenous mineralized extracellular matrix termed the “cement line.” Therefore, this matrix mechanically interlocks with the complex three-dimensional nanotopography of the bone surface created by the osteoclastic bone resorption, establishing the interface between and the anchorage of the new to the old bone. Subsequently, osteoblasts secrete collagen fibers that become organized, become mineralized, and bond to the cement line. Thus, the anchorage of the new to the old bone is mechanical (Figure 3) [7, 46].

The process of formation of new bone by contact osteogenesis on the implant surface during peri-implant bone wound healing is similar to the natural process of bone remodeling described above. Therefore, an implant surface microtopography which mimics the three-dimensional configuration of a bone surface immediately after osteoclastic bone resorption will promote new bone formation around the implant and ultimately its osseointegration and anchorage. Thus, modifying the implant surface to exhibit a three-dimensional complex topography on the micrometer and nanometer scales will be conducive to and inducive of the establishment of a “cement line” and the formation of bone which will interdigitate and interlock with the implant surface, ultimately promoting mechanical anchorage of implant to bone (Figure 2) [1, 7, 46].

## 5. Micrometer-Scale Surface Topography and Osseointegration

Moderately rough implant surfaces on the micrometer scale induce cells, particularly platelets, to produce and secrete biological mediators in the peri-implant microenvironment. These biological mediators attract differentiating osteogenic cells to the implant surface and promote the adhesion and stabilization of the blood clot and the formation of a fibrin matrix which acts as an osseoconductive scaffold for the migration of differentiating osteogenic cells that on contact with the implant surface will form bone, enhancing the mechanical stability of the implant during the initial phase of peri-implant bone wound healing (Figure 1) [7, 47].

Moderately rough implant surfaces with a complex microstructural topography have the capacity to induce and to regulate the expression of specific integrin subunits on the cell membrane of osteoblasts that are in contact with the implant. In turn, bone matrix proteins interact with these integrins, mediating osteoblast activity [28, 33]. During peri-implant bone wound healing, *α*_2_ *β*_1_ integrin signaling of mature osteoblasts stimulates production of angiogenic factors including vascular endothelial growth factor and fibroblast growth factor in an autocrine manner, thus mediating neoangiogenesis (Figure 1) [6].

It appears that in the early stages of peri-implant bone wound healing both the adhesion and subsequent stabilization of the blood clot in contact with the implant and the trabecular density of the newly formed woven bone are greater around implants with moderately rough surfaces of a high level of energy/wettability [7, 48]; and the remodelling of the newly formed woven bone into lamellar bone is more efficacious [48]. Implant surfaces that exhibit high energy/wettability induce differentiation of osteogenic cells, bringing about an increase in bone-to-implant contact ratio and an increase in the bonding strength of the bone-to-implant interface (Figure 3) [4, 6, 7].

## 6. Nanometer-Scale Surface Topography and Osseointegration

Cells, including osteogenic cells, interact with extracellular matrices or with biomaterial surfaces of nanometer topographic dimensions [49]. For example, the nanotopography of a biomaterial surface may bring about alterations in cell shape by interacting with cellular filopodia. Changes in cell shape may in turn influence cell differentiation [49]. It has been demonstrated that the nanostructural topography of a biomaterial has the capacity to direct the differentiation of the mesenchymal cell towards an osteogenic lineage and to direct differentiation of mesenchymal cells that have already become osteoprogenitor cells towards an osteoblastic phenotype [50–52].

Osteogenic cells in contact with a moderately rough titanium implant with nanostructural topography of their surface are predominantly polygonal with numerous filopodia extending across the ridges of the nanopeaks [51, 53]. The micro- and nanometer peaks and valleys of the implant surface consequently affect the organization of the cytoskeleton and of the intracellular transduction signaling pathways [11, 33].

As discussed above, ECM proteins bind to designated cell surface receptors termed integrins. The interaction between integrins and their ligands mediates cell adhesion to either extracellular matrices or biomaterials, stimulating intercellular signaling pathways which modulate cell function. It is believed that nanostructures of the biomaterial surface have the capacity to influence the nature of the interaction between the integrin and ECM proteins and therefore the cellular activity [47].

It is unknown whether the osteogenic events described above are directly induced by the nanostructural topography of the titanium surface or are indirectly brought about by the serum and local tissue fluid proteins that are selectively adsorbed and/or by the nanostructure-induced increase in surface energy/wettability [49]. Most probably all these mechanisms operate synergistically in bringing about an osteogenic effect [53].

## 7. Summary

Chemically modified rough implant surfaces with high energy/wettability and with a complex topography on nano- and micrometer scales promote healing processes in the microvoids between the implant and the bone at the osteotomy site resulting in enhanced osseointegration.
